# Building Osteogenic Microenvironments with a Double-Network Composite Hydrogel for Bone Repair

**DOI:** 10.34133/research.0021

**Published:** 2023-01-10

**Authors:** Jiaying Li, Jinjin Ma, Qian Feng, En Xie, Qingchen Meng, Wenmiao Shu, Junxi Wu, Liming Bian, Fengxuan Han, Bin Li

**Affiliations:** ^1^ State Key Laboratory of Radiation Medicine and Protection, School of Radiation Medicine and Protection and Interdisciplinary Sciences (RAD-X), Collaborative Innovation Center of Radiation Medicine of Jiangsu Higher Education Institutions, The First Affiliated Hospital, Suzhou Medical College, Soochow University, Suzhou, Jiangsu 215006, China.; ^2^ Orthopedic Institute, Department of Orthopaedic Surgery, The First Affiliated Hospital, Suzhou Medical College, Soochow University, Suzhou, Jiangsu, China.; ^3^ Key Laboratory of Biorheological Science and Technology, Ministry of Education, College of Bioengineering, Chongqing University, Chongqing 400044, China.; ^4^ Department of Biomedical Engineering, University of Strathclyde, Glasgow G1 1QE, UK.; ^5^ School of Biomedical Sciences and Engineering,South China University of Technology, Guangzhou International Campus, Guangzhou 511442, China.; ^6^ National Engineering Research Center for Tissue Restoration and Reconstruction, South China University of Technology, Guangzhou 510006, China.; ^7^ Guangdong Provincial Key Laboratory of Biomedical Engineering, South China University of Technology, Guangzhou 510006, China.; ^8^ Key Laboratory of Biomedical Materials and Engineering of the Ministry of Education, South China University of Technology, Guangzhou 510006, China.

## Abstract

The critical factor determining the in vivo effect of bone repair materials is the microenvironment, which greatly depends on their abilities to promote vascularization and bone formation. However, implant materials are far from ideal candidates for guiding bone regeneration due to their deficient angiogenic and osteogenic microenvironments. Herein, a double-network composite hydrogel combining vascular endothelial growth factor (VEGF)-mimetic peptide with hydroxyapatite (HA) precursor was developed to build an osteogenic microenvironment for bone repair. The hydrogel was prepared by mixing acrylated β-cyclodextrins and octacalcium phosphate (OCP), an HA precursor, with gelatin solution, followed by ultraviolet photo-crosslinking. To improve the angiogenic potential of the hydrogel, QK, a VEGF-mimicking peptide, was loaded in acrylated β-cyclodextrins. The QK-loaded hydrogel promoted tube formation of human umbilical vein endothelial cells and upregulated the expression of angiogenesis-related genes, such as *Flt1*, *Kdr*, and *VEGF*, in bone marrow mesenchymal stem cells. Moreover, QK could recruit bone marrow mesenchymal stem cells. Furthermore, OCP in the composite hydrogel could be transformed into HA and release calcium ions facilitating bone regeneration. The double-network composite hydrogel integrated QK and OCP showed obvious osteoinductive activity. The results of animal experiments showed that the composite hydrogel enhanced bone regeneration in skull defects of rats, due to perfect synergistic effects of QK and OCP on vascularized bone regeneration. In summary, improving the angiogenic and osteogenic microenvironments by our double-network composite hydrogel shows promising prospects for bone repair.

## Introduction

Bone is a crucial and multifunctional organ. Orthopedic disease is a common complication that can be caused by trauma, bone tumor, and other pathological factors [1]. After injury, bone has inherent capabilities for regeneration, but fails to achieve self-healing if the size of defects is large. Currently, autografts and allografts are widely considered as the gold standard for bone regeneration, but the high risks of infection and rejection, limited supply, and donor site morbidity severely limit the efforts of this bone therapy [2,3]. In recent decades, bone tissue engineering has emerged as a promising strategy for bone reconstruction [3,4]. In bone tissue engineering, biomaterial can act as a temporary matrix that provides a favorable microenvironment and suitable structure for cell growth and bone formation [5]. Furthermore, the combination of scaffolds and growth factors or drugs also shows excellent osteoinductive activity [3,6]. However, these materials are far from ideal candidates for guiding bone regeneration due to their deficient insufficient angiogenesis and osteogenic inductivity.

Bone is a highly vascularized tissue, and the skeletal vascular network plays a key role in bone development and repair [7–10]. After bone injury, the invasion of growing blood vessels is an important step in the process of osteogenesis [8,11,12]. Apart from exchanging nutrients, gases, hormones, electrolytes, and wastes, blood vessels are also involved in bone development, regeneration, and hematopoiesis [13]. Various studies have shown the importance of neovascularization for bone repair and regeneration [14–17]. After bone injury, the ingrowth of new blood vessels is important for the invasion of mesenchymal stem cells, which then promote osteogenic differentiation of cells and bone regeneration [18]. Furthermore, the blood vessels at injured sites are ruptured, and the blood flow in the total bone is reduced by 50% after bone injury, leading to a marked drop in oxygen tension inside the medullary [19]. Subsequently, the hypoxic microenvironment is formed at bone injury sites. The hypoxic microenvironment of bone injury could affect the genetic expression of osteoprogenitor cells [20], thus inhibiting osteogenic differentiation of cells and delaying bone repair. Therefore, developing an angiogenic microenvironment by the scaffolds that accelerates angiogenesis is an urgent need for bone formation.

Angiogenic growth factors are expressed by various cells and can combine with their respective receptors, inducing the migration and invasion of endothelial cells, and resulting in the formation of new blood vessels [18]. Therefore, the sustained delivery of angiogenic growth factors, such as the vascular endothelial growth factor (VEGF), is crucial to angiogenesis [21,22]. A scaffold that releases the basic fibroblast growth factor (bFGF) also effectively promotes the angiogenesis of periodontal ligament stem cells and eventually facilitates satisfactory periodontal bone regeneration, further demonstrating the key role of angiogenesis for bone formation [23]. Moreover, VEGF also promotes recruitment of cells to bone injury sites, participating in the process of bone repair, such as hematoma, endochondral bone formation, and intramembranous bone formation [22,24,25]. However, the use of growth factor proteins has several inherent disadvantages, such as lower stability, immunogenicity, and easy denaturation. Therefore, using short peptides for respective receptors instead of whole recombinant proteins will elude various side effects and minimize costs [26]. As a VEGF-mimicking peptide, QK is a synthetic 15-amino-acid peptide containing the 17–25 alpha region of the VEGF165 protein. Based on the region of VEGF, QK binds and activates both KDR (VEGFR-2) and Flt-1 (VEGFR-1) receptors, and further activates the same angiogenic response as VEGF does [27,28]. In a previous study, QK could covalently bind to a poly(ethylene glycol) hydrogel, enhancing vessel branch points and vessel density and promoting angiogenesis in tissue-engineered constructs [27]. Furthermore, it could also graft to polyamide 66 polymer chains to strengthen angiogenesis and bone formation [29]. Therefore, a scaffold that can continuously deliver QK may act as an excellent candidate to promote angiogenesis for bone regeneration.

In addition to angiogenesis, the bone tissue scaffolds with superior osteogenic microenvironment are more conducive to osteogenic differentiation of bone marrow mesenchymal stem cells (BMSCs) [30,31]. Bone is a kind of hard connective tissue made of cells, fibers, and the matrix, which contains abundant inorganic minerals, such as calcium phosphate (CaP) apatite crystals [32]. Octacalcium phosphate (OCP) is an acidic CaP and shows higher osteoconductive properties compared to hydroxyapatite (HA) and Ca-deficient HA or other precursors to HA [33,34]. Under physiological pH, OCP tends to be transformed into HA and promotes osteogenic differentiation of stem cells. In addition, OCP can be biodegraded through direct resorption by osteoclast-like cells, which have faster degradation than HA [34]. Various studies have demonstrated the excellent osteoinductive activity of OCP. For example, Saito et al. [35] found that OCP stimulated early osteocyte differentiation in the bone matrix. In addition, Amann et al. [36] fabricated sponge-like chitosan-collagen-OCP scaffolds, and OCP at the top of the bony layer showed excellent osteogenic induction. Hence, the combination of OCP and bone tissue scaffold can realize better osteoinductive activity for bone regeneration.

**Fig. 1.**
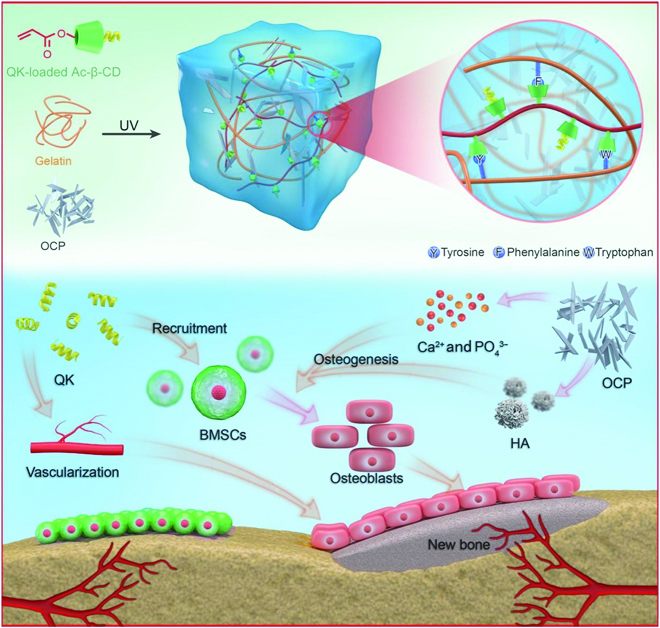
Schematic illustration of double-network composite hydrogels that build an angiogenic and osteogenic microenvironment for bone repair. The QK released from the composite hydrogel recruits BMSCs to the sites of bone injury and directly promotes angiogenesis, then Ca^2+^ and HA induce osteogenic differentiation of BMSCs and promote bone formation.

In this study, we added acrylated β-cyclodextrins (Ac-β-CD) to gelatin to fabricate a double-network hydrogel through the chemical crosslinking of Ac-β-CD and physical crosslinking by host–guest interactions between CD and gelatin. To build an angiogenic and osteogenic microenvironment for bone repair, QK, which is a VEGF-mimicking peptide, and OCP, which is an HA precursor and could release calcium ions (Ca^2+^), were introduced into the double-network hydrogel. The property of the double-network hydrogel and release behavior of QK will be characterized. Both in vitro angiogenesis and osteogenesis abilities of the composite hydrogels will be evaluated. The in vivo angiogenesis ability will be detected in the subcutaneous implantation experiment. The repair of calvarial defects of rats by the double-network composite hydrogel will be evaluated (Fig. 1).

## Results

### Characterizations of the double-network composite hydrogels

**Fig. 2.**
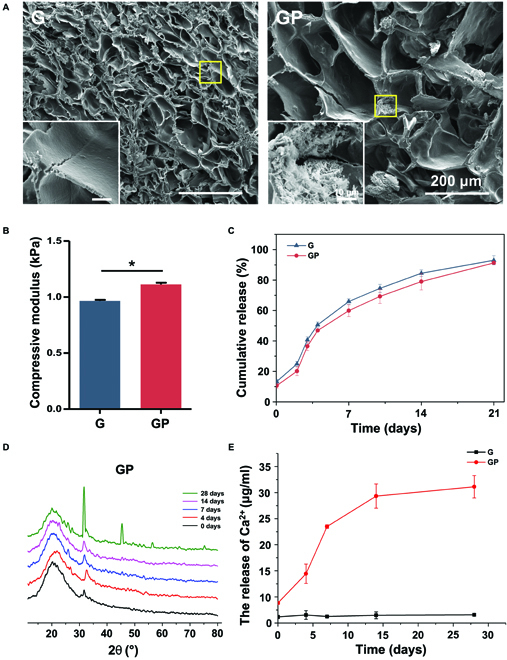
Characteristics of hydrogels. (A) SEM images of the cross section of hydrogels. (B) Compressive modulus of hydrogels. (C) QK release curves of hydrogels. (D) XRD measurement of the GP composite hydrogel. (E) Ca^2+^ release curves of hydrogels. *P < 0.05.

To prepare the composite hydrogels, we added gelatin and prepared Ac-β-CD into phosphate buffered saline (PBS) solution and completely dissolved it at 37 °C, after which the initiators 2-hydroxy-4′-(2-hydroxyethoxy)-2-methylpropiophenone (I2959) and OCP were added. After ultraviolet (UV) crosslinking, the composite hydrogels were obtained. As shown in Fig. 2A, the obtained hydrogels of G and GP both exhibited interconnected macroporous structures, and the pore size was increased with the OCP addition. The OCP that showed sheet structure (Fig. S1A) was distributed uniformly in composite hydrogels. As expected, the mechanical property of GP was increased to 1.11 ± 0.02 kPa (Fig. 2B). Moreover, as shown in Fig. S2, G and GP composite hydrogels could load QK, and the QK-loading efficiency of G and GP hydrogels was 39.9% ± 6.2% and 49.6% ± 15.8%, respectively. Figure 2C shows that about 92.99% ± 3.02% and 91.24% ± 1.03% of QK were released from G and GP within 21 days, respectively. The drug release tests present that the release rate of QK from G and GP was parallel, exhibiting no significant difference.

To observe whether OCP can be converted into HA, G and GP composite hydrogels were immersed into PBS for 28 days, and the morphology of G and GP was observed. Scanning electron microscopy (SEM) images show that a large number of calcium minerals emerged in the GP composite hydrogel (Fig. S3). To determine the produced calcium minerals, the x-ray diffractometer (XRD) measurement was performed. The result shows that OCP in the GP composite hydrogel barely converted into HA after 14 days while producing a large amount of HA after incubation in PBS for 28 days (Fig. 2D). Moreover, the GP composite hydrogel sustained released Ca^2+^; the cumulative release of Ca^2+^ reached 31.1 ± 2.1 μg ml^−1^, which was significantly higher than that of the G composite hydrogel (6.6 ± 0.2 μg ml^−1^) (Fig. 2E). The results of the swelling property reveal that the G composite hydrogel showed a higher swelling ratio than GP after 24 h. The results of degradation measurement demonstrate that the weight of G and GP barely changed in PBS solution after 9 days (Fig. S4). Therefore, OCP may be an excellent candidate for bone repair due to its conversion into HA and sustained Ca^2+^ release properties.

### In vitro biocompatibility tests of the composite hydrogels

**Fig. 3.**
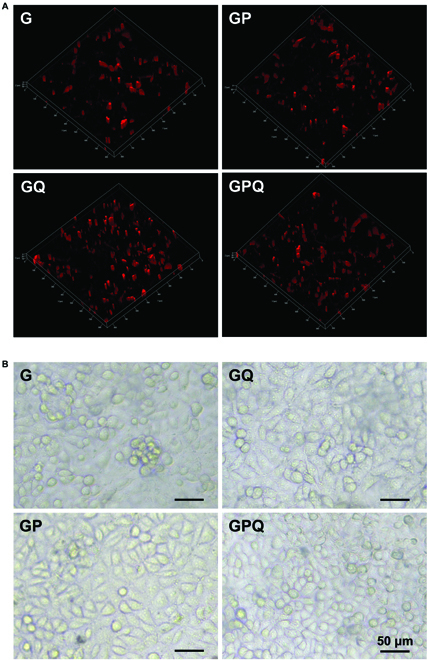
Growth of BMSCs and HUVECs on the surface of hydrogels. (A) Dil-labeled BMSCs cultured on the hydrogel surface after 3 days. (B) Morphology of HUVECs on the hydrogel surface after 2 days.

Rat BMSCs were used to determine the biocompatibility of G, GP, and QK-loaded hydrogels. The addition of OCP in G showed negligible cytotoxicity, indicating that GP composite hydrogels had good biocompatibility and could fabricate cell growth (Fig. 3A and B). The results of CCK8 demonstrate that after loading QK, GQ and GPQ also had no toxic effects and even promoted cell proliferation, and the number of cells on GQ and GPQ samples was higher (Fig. S5). Both the BMSCs and human umbilical vein endothelial cells (HUVECs) could maintain the high viability on all hydrogels, which provided basic conditions for further application.

### Characterizations of cell migration

**Fig. 4.**
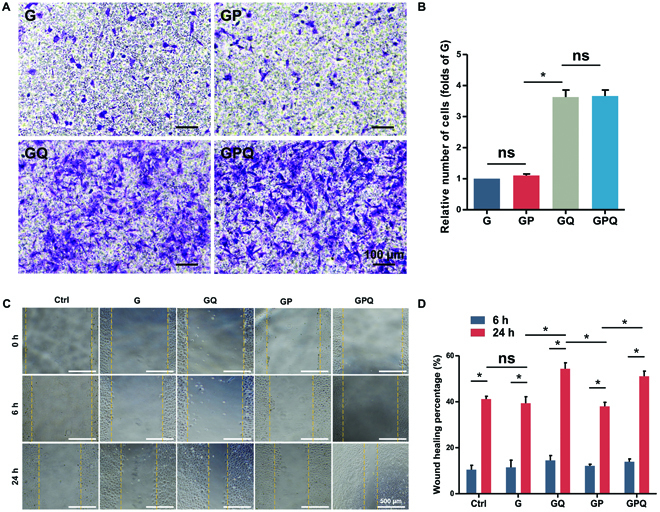
Characterizations of cell migration. (A) Crystal violet staining of BMSCs that migrated to the lower chamber of the transwell plate after 24-h culture. (B) Manual random counting in 5 random fields under 100× magnification. (C) Wound-healing assay of a monolayer of BMSCs after 0, 6, and 24 h. Wound boundary was marked with a yellow dashed line. (D) Calculation of wound closure area after 6 and 24 h. *P < 0.05. ns, no significant difference.

To determine the cell recruitment effect of the hydrogels, we performed the transwell assay in vitro. The results show that G and GP hydrogels had a faint ability to recruit cells, and the cells in G and GP groups were significantly lower. In contrast, the cell recruitment effect of hydrogels was significantly enhanced after loading QK. The recruited cells in GQ were about 3.5-fold those of G hydrogels, and GPQ composite hydrogels likewise showed an excellent cell recruitment effect (Fig. 4A and B). Furthermore, we carried out a cell scratch assay to further verify the cell recruitment effect of hydrogels. After 6 h of culture, we found that the wound-healing percentage of all groups reached about 10%, exhibiting no significant difference. However, the wound-healing percentage of G and GP hydrogels reached about 40% after 24 h of culture, which was similar to the Ctrl (C) group and showed no evident cell migration effect. In addition, the wound-healing percentage of GQ and GPQ hydrogels reached approximately 57% and 50%, respectively, indicating that QK significantly promoted cell migration (Fig. 4C and D) and exhibited excellent cell recruitment effects for tissue repair.

### Characterizations of vascularization

**Fig. 5.**
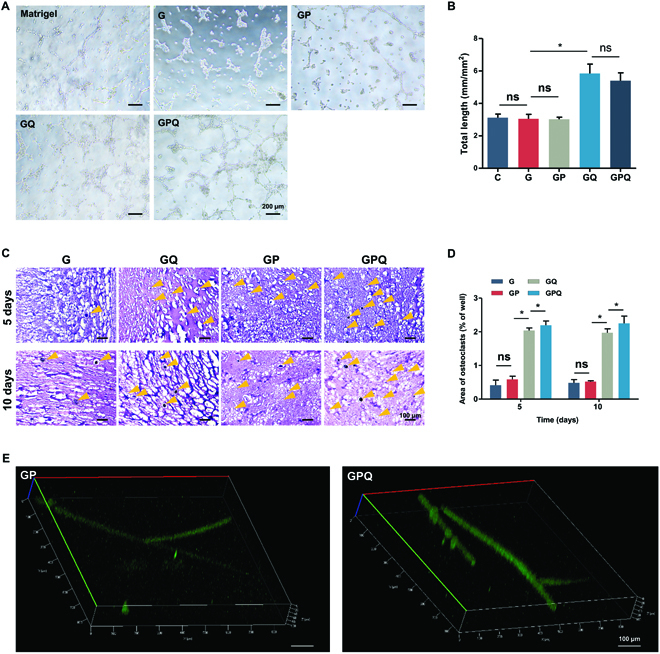
Characterizations of vascularization. (A) Tube formation assay of HUVECs in different groups. (B) Quantification of average parameters of tube formation. (C) H&E staining of hydrogels implanted into subcutaneous tissue of rat after 5 and 10 days. Blood vessels were marked with yellow arrowheads. (D) Quantification of the area of blood vessel formation. (E) Fluorescein isothiocyanate labeling of blood vessels formation in hydrogels that were implanted into subcutaneous tissue of rat after 10 days. *P < 0.05. ns, no significant difference.

Angiogenesis was evaluated by the tube formation assay on the Matrigel. After 4 h, a primary vascular-like network structure was induced with QK-loading groups (GQ and GPQ), and the total length of the formed tubular structures by GQ and GPQ composite hydrogels reached about 5.8 and 6 mm/mm^2^, respectively, which was significantly longer than G and GP hydrogels (G: 3 mm/mm^2^, GP: 3.1 mm/mm^2^) (Fig. 5A and B). Moreover, the hematoxylin–eosin (H&E) staining of hydrogels that were implanted into subcutaneous tissue of rats showed that GQ and GPQ composite hydrogels significantly promoted angiogenesis. Five days after implantation, the area of new blood vessels in GQ and GPQ groups was about 2.0% ± 0.1% and 2.2% ± 0.2%, respectively, which was higher than G (0.4% ± 0.2%) and GP (0.6% ± 0.1%). The area of new blood vessels in GQ and GPQ groups reached about 2.0% ± 0.2% and 2.3% ± 0.3% after 10 days of implantation, demonstrating the excellent property of QK for angiogenesis (Fig. 5C and D). We also visualized the blood vessel formation inside the hydrogels after intravenous injection of solution containing fluorescein isothiocyanate–dextran. After 10 days of implantation, we found that the blood vessel formation in GPQ composite hydrogels was larger in size and had distinct branches, while the blood vessels in GP group were very small and with few branches (Fig. 5E).

**Fig. 6.**
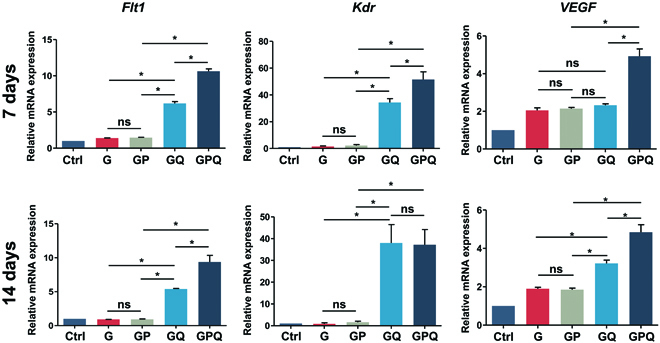
Expression of angiogenesis-related genes of BMSCs cultured with hydrogels after 7 and 14 days. *P < 0.05. ns, no significant difference.

To further examine the in vitro angiogenic potential of hydrogels, real-time polymerase chain reaction (PCR) was carried out to evaluate the expression of angiogenesis-related genes. The results show that QK-loading hydrogels (GQ and GPQ) could upregulate the expression of *Flt1*, *Kdr,* and *VEGF*, further indicating the excellent angiogenic activity produced by QK (Fig. 6).

### Characterizations of in vitro osteoinductive activity

**Fig. 7.**
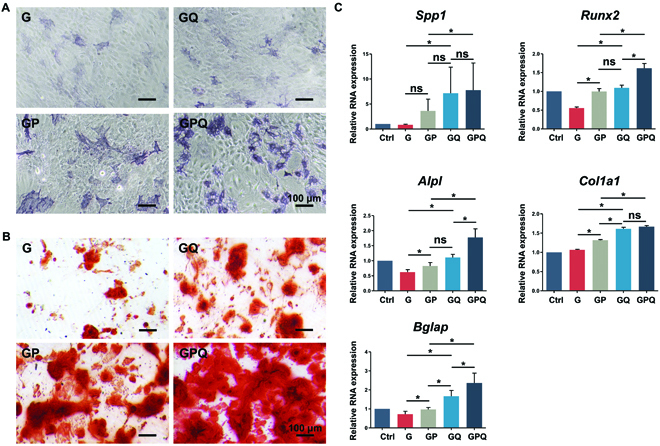
Characterizations of in vitro osteogenic differentiation of BMSCs. (A) ALP staining of BMSCs in different groups after 7 days. (B) Alizarin red staining of BMSCs in different groups after 21 days. (C) Expression of osteogenesis-related genes of BMSCs cultured with hydrogels: *Spp1*, *Runx2*, and *Alpl* after 7 days; *Col1a1* and *Bglap* after 21 days. *P < 0.05. ns, no significant difference.

To determine the in vitro osteogenic activity of BMSCs cultured with hydrogels, alkaline phosphatase (ALP) staining, alizarin red staining, and the expression of osteogenesis-related genes were tested. The results show that G and GQ hydrogels could slightly upregulate the expression of ALP, while GP and GPQ composite hydrogels significantly promoted ALP expression (Fig. 7A and Fig. S6A). After 21 days, plenty of calcium nodules were produced in the GPQ group. GP composite hydrogels also produced abundant calcium deposition, which was more than G and GQ hydrogels (Fig. 7B and Fig. S6B).

Quantitative PCR results show that GPQ hydrogels obviously promoted the expression of osteogenic genes, such as *Alpl*, *Spp1*, and *Runx2*. Compared with G hydrogels, GP and GQ upregulated the expression of *Spp1* and *Runx2*. After 21 days, GPQ composite hydrogels could also promote the expression of *Bglap* and *Col1a1*, demonstrating the excellent effects for inducing osteogenic differentiation of BMSCs (Fig. 7C).

### Promotion of in vivo bone formation by the double-network composite hydrogels

**Fig. 8.**
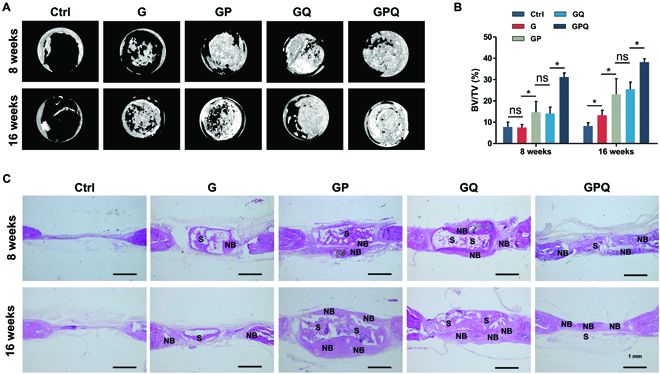
Characterizations of in vivo bone repair by double-network composite hydrogels. (A) Micro-computed tomography images at 8 and 16 weeks. (B) Bone volume fraction. (C) H&E staining of calvarial critical-sized defects repaired by hydrogels. S, scaffold; NB, new bone. *P < 0.05. ns, no significant difference.

To verify the bone repair ability of our fabricated hydrogels, a rat cranial defect model was introduced. After 8 weeks of surgery, there was barely observable new bone formed in Ctrl and G groups. Less new bone was formed in GP and GQ groups, while obvious new bone was formed in the GPQ group, demonstrating a significant synergistic effect of OCP and QK for bone repair (Fig. 8A). The percent bone volume (BV/TV) indicates that the new bone in the GPQ group reached about 30%. The new bone repaired by GPQ completely filled the whole damaged area, and the BV/TV value reached about 37% at 16 weeks after operation (Fig. 8B). However, the bone mineral density of samples repaired by composite hydrogels exhibited no difference (Fig. S7). H&E staining shows that new bone formation and bone maturation in the GPQ group were better than those in other groups (Fig. 8C).

### Discussion

Bone tissue plays a key role in providing a framework for attachment of muscles and other tissues, protecting internal organs from damage, and maintaining calcium homeostasis and acid/base buffering [37,38]. Although bone has a high self-healing capacity to realize regeneration, it is unable to achieve self-regeneration if the defects are larger than critical-size defects. Currently, bone tissue engineering is considered an alternative solution for repairing large defects.

There have been plenty of scaffolds that show excellent osteoinductive activity, but these materials are far from ideal candidates for guiding bone regeneration due to their insufficient angiogenesis and osteogenesis. Herein, we attempt to build a favorable osteogenic microenvironment by combining VEGF-mimetic peptide and HA precursor with the hydrogels to promote both vascularization and osteogenesis capability. Compared with the other vascularization strategies, the method of loading QK is simple but has the same function as VEGF. Moreover, QK could recruit BMSCs to bone injury sites. To build an osteogenic microenvironment, the OCP, an HA precursor, is introduced into the hydrogels. Furthermore, OCP in the composite hydrogels could transform into HA and release calcium ions, which would be involved in bone regeneration. The double-network composite hydrogel integrated QK and OCP showed obvious osteoinductive activity.

Hydrogel, a tissue engineering scaffold with a very high water content, can simulate extracellular matrix to provide a suitable environment for cell survival, proliferation, and differentiation [39,40]. Moreover, hydrogel can also fill the damaged area perfectly and promote bone repair [41]. In this study, Ac-β-CD and OCP were added into gelatin to fabricate double-network composite hydrogels after UV-initiated polymerization. The obtained hydrogels exhibited an interconnected macroporous structure, which was beneficial to nutrient exchange and metabolic waste removal. As previously reported [42], G hydrogels showed excellent Young’s modulus due to photo-crosslinking [43]. Native gelatin can spontaneously form hydrogels at low temperatures (<30 °C) due to physical crosslinking; however, the triple helix of gelatin is disrupted at temperatures above 30 °C, showing extremely labile performance and limiting their application for tissue engineering [44]. In this study, the added β-CD does 2 things. On the one hand, it can form host–guest complexation with aromatic residues of gelatin (e.g., phenylalanine, tyrosine, and tryptophan) in the hydrogels, which could enhance the mechanical performance under physiological conditions and produce a stable hydrogel. On the other hand, it acts as the drug carrier of QK due to the inclusion complex formation of CD with a wide array of small molecules [45,46].

Many factors affect bone healing after injury, such as vascularization [47], inflammation [48], osteoporosis, and infection [49,50]. Herein, we focus on the angiogenic and osteogenic factors in the microenvironment of bone injury. Bone is a highly vascularized tissue, and various studies demonstrated that angiogenesis is closely associated with bone regeneration [13,14,51]. When bone is fractured, the blood vessels are damaged, hematoma forms, and inflammatory cells migrate into the fracture site. The ingrowth of new blood vessels is essential for the formation of a soft callus. The soft callus is converted into a rigid, calcified hard callus. Further mineralization and remodeling processes within the callus eventually lead to the repair of the damaged bone. Blood vessel formation is a necessary part of bone repair due to its effects of providing adequate nutrients, growth factors, and oxygen, and transferring waste products from the damaged area. Moreover, blood vessels can also maintain cell viability during the process of bone repair [52].

Because of the great importance of vascularization in both bone formation and in bone healing, it is clear that this aspect must be taken into account in bone tissue engineering. Qiu et al. fabricated periosteal extracellular matrix hydrogel for bone regeneration. The periosteal extracellular matrix hydrogel showed excellent potential for migration and development of blood vessels, indicating the importance of vascularization for bone regeneration [53]. In addition, Jin et al. [54] used poly(lactate-*co*-glycolate)/fish collagen/nano-hydroxyapatite (PFCH) fibrous membranes for bone repair and found that latticed PFCH membrane exhibits optimal function in inducing angiogenesis. Apart from bioactive materials that promote angiogenesis, numerous scaffolds can also realize bone regeneration by delivering angiogenic growth factors to the sites of bone injury. For example, Zhou et al. [55] loaded bFGF into methacrylate gelatin to simulate angiogenic signaling, realizing evident angiogenic effects for bone regeneration by continuously releasing a high concentration of bFGF at bone injury sites. Herein, we combined QK and hydrogel to engineer QK-loaded scaffolds due to the inclusion complex formation of Ac-β-CD with QK. Compared with the other vascularization strategies, the method of loading QK is simple but has the same function as VEGF. Moreover, the reversible nature of host–guest interactions also allows the hydrogel to dynamically modulate the release of encapsulated drugs on demand. In addition, QK could recruit BMSCs to bone injury sites. Compared to G and GP, the obtained GQ and GPQ hydrogels continuously release QK, observably promoting HUVECs to form tubular structures. Various studies also accelerate bone repair by delivering VEGF [21,56,57], initiating angiogenesis by binding to the transmembrane VEGF receptors [58]. However, VEGF shows low stability, easily denatures, and loses bioactivity. As a VEGF-mimicking peptide, QK can avoid the side effects and minimize costs, while maintaining excellent angiogenesis capability. Therefore, our fabricated GQ and GPQ hydrogels could also significantly upregulate the expression of *VEGF* and VEGF receptors, such as *Flt1* and *Kdr*, exhibiting perfect effects for vascularization. Bone repair is a long process, and early vascularization is important for bone repair. In this study, QK is released about 90% within 21 days, which means it can promote early vascularization. Moreover, the rapid release of QK in the early stage also recruits cells to help bone repair. Therefore, the composite hydrogel loading QK could promote bone repair, though the QK might not be involved in the whole process of bone repair.

Osteogenesis is the main endeavor for bone tissue scaffolds. G hydrogels could act as a good candidate for cell adhesion and proliferation and show evident biocompatibility. However, G hydrogels lack osteoinductive activity and thus delay bone healing. Compared with other orthophosphates, OCP is regarded as the precursor phase of apatite formation, which can be converted to the apatite form under physiological conditions [59]. Thus, it is viewed as the most promising candidate for bone tissue regeneration [60]. HA is the major inorganic component in bone tissue and has been widely used to promote bone regeneration owing to its strong enhancement of osteogenic differentiation [61,62]. In this study, we found that OCP significantly converted into HA and released Ca^2+^. After addition into G hydrogels, the obtained GP composite hydrogel significantly induced osteogenic differentiation of BMSCs. Moreover, the GPQ further increased the osteoinductive activity of BMSCs and exhibits good potential for bone repair. After implantation in rat cranial defects, our fabricated GP composite hydrogels showed excellent osteoinductive properties for bone regeneration. As expected, the GQ composite hydrogel could also form new bone owing to its good angiogenesis. More importantly, the engineered GPQ composite hydrogel formed abundant new bone and was almost full of the defects, indicating that the combination of QK and OCP could synergistically promote new bone formation and bone maturation by building an angiogenic and osteogenic microenvironment. The blood vessels play a crucial role in bone repair. In this study, the composite hydrogel builds a favorable microenvironment for angiogenesis by constantly releasing QK. The OCP in the hydrogels could release Ca^2+^ and be transformed into HA, which builds the osteogenic microenvironment to induce osteogenic differentiation of BMSCs and promotes bone formation. The angiogenic microenvironment and osteogenic microenvironment synergically promoted bone regeneration.

Overall, it is important to build a favorable microenvironment by the scaffolds for bone repair. In the future, we can build a scaffold with stronger vascularization and bone regeneration function. In addition, there are many other factors that also affect bone regeneration, such as inflammation, osteoporosis, and infection. Therefore, it is important to develop the corresponding scaffolds according to different bone injuries, to establish a suitable microenvironment that is conducive to cell survival, proliferation, and differentiation and bone regeneration.

A double-network composite hydrogel containing an HA precursor and a VEGF-mimicking peptide has been developed in this study. This composite hydrogel could constantly release Ca^2+^ and QK, which build a favorable microenvironment for angiogenesis and osteogenesis. This hydrogel promoted in vitro tube formation, upregulating *VEGF* and VEGF receptors and in vivo blood vessel formation. In addition, the hydrogel enhanced in vitro osteogenesis according to the results of ALP staining, alizarin red staining, and expressions of osteogenesis-related genes. The results of animal experiments show that the composite hydrogel enhanced bone formation in calvarial defects of rats, which shows perfect synergistic effects of QK and OCP for vascularized bone regeneration. It is concluded that the strategy of improving the angiogenic and osteogenic microenvironment by our double-network composite hydrogel shows promising prospects for vascularized bone repair.

## Materials and Methods

### Materials

Gelatin (type A, from porcine skin, isoelectric point: 7–9, Cat. No. G1890-500G, Sigma), triethyl amine, and I2959 were purchased from Sigma. QK (KLTWQELYQLKYKGI, 99% purity) was provided by Shanghai Qiangyao Biological Technology Co., Ltd. (Shanghai, China). β-CD and OCP were bought from Shanghai Aladdin Biochemical Technology Co., Ltd. (Shanghai, China). Dimethyl formamide was purchased from Fisher Scientific. 1,1′-Dioctadecyl-3,3,3′,3′-tetramethylindocarbocyanine perchlorate (Dil) was purchased from KeyGEN BioTECH (China). Alpha’s modified Eagle’s medium (α-MEM), Dulbecco's Modified Eagle Media:Nutrient Mixture F-12 (1:1) (DMEM/F12 [1:1]) medium, and PBS were provided by Hyclone (USA). Fetal bovine serum (FBS) was purchased from Gibco (USA).

### Preparation of Ac-β-CD

Ac-β-CD was fabricated according to a previous study [42]. In brief, 10 g of β-CD was added to the solution, which contained 150 ml of dimethyl formamide and 7 ml of triethyl amine. The mixture was stirred and cooled down to 0 °C; then, 5 ml of acrylic anhydride was added into the above solution and stirred for 12 h. The reaction mixture was filtrated and then concentrated to approximately 20 ml by vacuum rotary evaporation. Then, the solution was dripped into 600 ml of acetone to precipitate the modified β-CD. The precipitate was washed with acetone and vacuum dried for 3 days.

### Preparation of the double-network composite hydrogels

To prepare composite hydrogels, gelatin (10% [w/v]) and Ac-β-CD (10% [w/v]) were added into PBS solution and then dissolved completely at 37 °C. The initiator I2959 was added at 0.05% (w/v). After photo-crosslinking by UV light (365 nm), the double-network hydrogel was obtained, termed G hydrogel. Then, the OCP (100 mg ml^−1^) was added to the above mixture, and double-network composite hydrogels (GP) were obtained after photo-crosslinking.

### Morphology of the composite hydrogels

The obtained composite hydrogels were dried via lyophilization. After being sputter-coated with gold using an Ion Sputter (SC7620, Quorum Technologies, Lewes, UK), the morphology of the samples was characterized by SEM (Quanta 250, FEI, Hillsboro, OR, USA).

### Mechanical tests

The mechanical property of cylindrical hydrogels (diameter, 4.5 mm; height, 5 mm) were tested (2 mm min^−1^) using a universal mechanical testing system (Shanghai Hengyi Precision Instrument Co., Ltd., Shanghai, China).

### Measurement of QK-loading and release

The drug loading and delivery behaviors of composite hydrogels were measured; the obtained G and GP composites were dispersed in QK solution (2 mg ml^−1^) and shaken at 37 °C for 12 h to obtain GQ and GPQ hydrogels. The loading rate of QK was measured via a bicinchoninic acid protein assay kit (Beijing ComWin Biotech Co., Ltd., Beijing, China) at the wavelength of 562 nm. At different time points, the suspension was collected and the release of QK from GQ and GPQ composite hydrogels was measured via a bicinchoninic acid protein assay kit at the wavelength of 562 nm.

### Calcium release tests

The property of calcium release from composite hydrogels was tested. A total of 100 μl of G and GP composite hydrogels was obtained after photo-crosslinking by UV light. Then, the G and GP composite hydrogels were soaked in 1 ml of PBS. At different time points, the suspension was collected, and the amount of calcium was tested by the calcium colorimetric assay kit.

### Swelling and degradation tests

The hydrogels were immersed into PBS solution. After 24 h, the weight of G and GP composite hydrogels was evaluated. The weight of the hydrogel after dehydration is *W*_D_. The weight of the hydrogel after swelling equilibrium is *W*_S_. The swelling rate of G and GP was calculated using the following formula:

$$\text{Swelling rate}\;(\%)=W_{S}/W_{D}\times 100\%$$

The hydrogels were immersed into PBS solution. The initial weight of the hydrogel is *W*_I_, and the final mass of the hydrogel is *W*_F_. The remaining mass of G and GP was measured after 1, 3, 5, 7, and 9 days.

$$\text{Remaining mass}\;(\%)=W_{F}/W_{I}\times 100\%$$

### X-ray diffraction

G and GP composite hydrogels were dried via lyophilization after testing calcium release. Then, the power of samples was analyzed by XRD (D8 Advance, Bruker, Karlsruhe, Germany) at 40 kV and 40 mA. Data were collected for 2*θ* ranging between 51 and 701 under CuKα radiation (*l* = 1.54056). The step size was 0.0161, and the residence time was 10 s.

### Measurements of biocompatibility

The biocompatibility of the hydrogels was evaluated using rat BMSCs and HUVECs. Rat BMSCs were labeled by Dil fluorescence dye (10 nM) after incubation for 20 min; then, the Dil-labeled BMSCs were seeded on hydrogels (1 × 10^4^ cells/well) in a 48-well plate. In each well of the tissue culture plate, 150 μl of hydrogel was placed. Hydrogels were soaked in α-MEM culture medium containing 10% FBS and 1% penicillin–streptomycin. BMSCs cultured on the surface of hydrogels were observed using a laser scanning confocal microscope after 3 days. HUVECs were directly seeded on hydrogels (150 μl) at a density of 1 × 10^4^ cells/well in a 48-well plate. Samples were incubated with DMEM/F12 (1 1) culture medium (10% FBS and 1% penicillin–streptomycin). The morphology of HUVECs cultured on hydrogels was observed by an inverted microscope after 2 days.

To measure whether the hydrogels promoted cell proliferation, BMSCs and HUVECs were seeded in a 24-well plate (5×10^3^ cells/well) and cultured with α-MEM and DMEM/F12 culture medium (10% FBS and 1% penicillin–streptomycin), respectively. After 12 h, 500 μl of extract liquid of hydrogels (600 μl of hydrogel immersed in 1 ml of cell culture medium) was added. The optical density value of samples at 450 nm was measured after incubating with CCK-8 working solution.

### Characterizations of cell migration

Rat BMSCs were seeded in the upper chamber of a 24-well transwell plate (Corning, NY, USA) at a density of 1 × 10^4^ cells/well. Then, the hydrogels (300 μl) were placed in the lower chamber with α-MEM culture medium containing 10% FBS and 1% penicillin–streptomycin. After 24 h, rat BMSCs on the upper chamber were taken away and then fixed with 4% paraformaldehyde. Rat BMSCs were treated with 0.1% crystal violet solution (Beyotime, Nanjing, China), and the cells migrated into the bottom chamber were counted in 5 random fields.

Rat BMSCs were seeded in a 12-well plate (2 × 10^5^ cells/well) and cultured with α-MEM culture medium (10% FBS and 1% penicillin–streptomycin). Upon reaching confluence, BMSCs were scraped to create a wound. Then, the culture medium was changed into 1 ml of extract liquid of hydrogels. The extract liquid of hydrogels was prepared by adding 600 μl of hydrogel in a 12-well plate and then adding 1 ml of cell culture medium. At each time point, 1 ml of extract liquid was taken out, and 1 ml of new medium was supplemented. Rat BMSCs were photographed using an inverted microscope after 6 and 24 h. The distances of wound closure were observed using the microscope and calculated by ImageJ software (National Institutes of Health, Bethesda, USA).

### In vitro and in vivo pro-vascularization properties

HUVECs were seeded on the surface of growth factor-reduced Matrigel (1.8 × 10^5^ cells/well) in a 12-well plate. After half an hour, 1 ml of extract liquid of hydrogels was added. The extract liquid of hydrogels was prepared by adding 600 μl of hydrogel in a 12-well plate and then adding 1 ml of cell culture medium. After 4 h, the morphology of HUVECs was observed using an inverted microscope, and the average parameters of tube formation in 6 random fields were quantified by ImageJ software.

Rat BMSCs were seeded in the lower chamber of a 6-well transwell plate (1 × 10^5^ cells/well). When the cell fusion degree reached 60%, the complete medium was removed and replaced by the osteogenic differentiation medium (10 mM β-glycerophosphate, 10 nM dexamethasone, and 50 μg/ml l-ascorbic acid 2-phosphate, Cyagen Biosciences Inc., Guangzhou, China). Then, 1 ml of hydrogel was placed in the upper chamber and immersed in the cell culture medium. Hydrogels were placed in the upper chamber and immersed in the cell culture medium (control group: α-MEM complete medium). The expression of the vascularization-related genes (*Flt1*, *Kdr*, and *VEGF*) was measured using real-time PCR. Primer sequences are listed in Table S1.

The hydrogels (5 mm in diameter and 1 mm in thickness) were implanted into the subcutaneous tissue of rats to evaluate vascularization. After 5 and 10 days, the samples were gathered and fixed with 4% paraformaldehyde solution and cut into 10-μm-thick histological frozen sections; H&E staining was performed to assess the vascularization of hydrogels in vivo. After 10 days, perfusion was performed with a solution containing a fluorescent agent. The solution was prepared by adding fluorescein isothiocyanate–dextran (Sigma) into 4% paraformaldehyde solution at a concentration of 50 mg/ml. Then, the samples were visualized by a laser scanning confocal microscope to evaluate the in vivo vascularization of hydrogels.

### Determination of in vitro osteogenesis ability

Rat BMSCs were seeded in the lower chamber of a 24-well transwell plate (5 × 10^4^ cells/well). Three hundred microliters of hydrogel (G, GP, GQ, and GPQ) was placed in the top chamber. The cells were cultured with α-MEM complete medium. When the cell fusion degree reached 60%, the medium was replaced by osteogenic differentiation medium. Samples were fixed with 4% paraformaldehyde and then incubated with ALP staining solution (Beyotime, China) after 7 days. The quantitative characterization of ALP was performed by the ALP assay kit (Beyotime, China). The images of samples were collected by an inverted microscope. After 21 days, alizarin red staining (Cyagen Biosciences) was performed. The quantitative characterization of alizarin red staining was performed by measuring the optical density value at 420 nm after incubating with perchloric acid (10%).

Rat BMSCs were seeded in the lower chamber of a 6-well transwell plate (1 × 10^5^ cells/well). When the cell fusion degree reached 60%, the medium was replaced by osteogenic differentiation medium. Then, 1 ml of hydrogel was placed in the upper chamber and immersed in the cell culture medium. The expression of the osteogenesis-related genes (*Alpl*, *Spp1*, *Runx2*, *Col1a1*, and *Bglap*) was determined by real-time PCR. The primer sequences are listed in Table S1.

### Animal experiment

The procedures followed the National Institutes of Health Guide for the Care and Use of Laboratory Animals and were approved by the Institutional Animal Care and Use Committee of Soochow University (ECSU-201700041). The calvarial defects in rats were used to determine the in vivo bone formation ability of hydrogels. The male Sprague–Dawley rats (8 weeks old) were administered an intraperitoneal injection of pentobarbital sodium (30 mg/kg, Sigma). After deep anesthesia, calvarial defects (5 mm in diameter and 1 mm in thickness) were made using a micro bone drill, and then the hydrogels (G, GP, GQ, and GPQ) were implanted into the defect sites. In the Ctrl group, no material was implanted into the defects. Skulls of rat were acquired at 8 and 16 weeks after surgery, respectively. Each group had 3 replicates. Samples were harvested and managed using Micro-CT (65 kV, 385 mA, 1 mm Al filter). Then, rat skulls were decalcified, sliced, and stained with H&E.

### Statistical analysis

Data were presented as mean ± standard deviation. Statistical analysis (GraphPad Software, USA) was evaluated using one-way analysis of variance followed by Tukey’s multiple comparisons to evaluate differences between the groups. A probability value (*P*) of less than 0.05 was considered statistically significant.

## Data Availability

Data supporting the findings of this study can be obtained from the corresponding author upon request.

## Supplementary Materials

Supplementary Materials Fig. S1. SEM image (A) and XRD measurement (B) of OCP. Fig. S2. QK loading efficiency of the composite hydrogels. **P* < 0.05. Fig. S3. SEM images of the cross section of the hydrogels immersed in PBS. Fig. S4. Swelling property (A) and mass remaining (B) of the composite hydrogels immersed in PBS. **P* < 0.05. Fig. S5. Cell proliferation of (A) HUVECs and (B) BMSCs on the surface of composite hydrogels. **P* < 0.05. Fig. S6. Quantitative analysis of ALP (A) and alizarin red (B) staining. **P* < 0.05. Fig. S7. Bone mineral density of regenerated tissues in the bone defects. Table S1. PCR primer sequences.
