# Supplementary material for: Building Osteogenic Microenvironments with a Double-Network Composite Hydrogel for Bone Repair
Source: Research (Wash D C). 2023 Jan 10;6:0021. doi: 10.34133/research.0021 (PMC10076009; doi:10.34133/research.0021)
Supplement: Supplementary Materials — Fig. S1. SEM image (A) and XRD measurement (B) of OCP. Fig. S2. QK loading efficiency of the composite hydrogels. *P < 0.05. Fig. S3. SEM images of the cross section of the hydrogels immersed in PBS. Fig. S4. Swelling property (A) and mass remaining (B) of the composite hydrogels immersed in PBS. *P < 0.05. Fig. S5. Cell proliferation of (A) HUVECs and (B) BMSCs on the surface of composite hydrogels. *P < 0.05. Fig. S6. Quantitative analysis of ALP (A) and alizarin red (B) staining. *P < 0.05. Fig. S7. Bone mineral density of regenerated tissues in the bone defects. Table S1. PCR primer sequences. [file research.0021.f1.docx]

Building Osteogenic Microenvironments with a Double Network Composite Hydrogel for Bone Repair

**Jiaying Li^1^, Jinjin Ma^2^, Qian Feng^3^, En Xie^2^, Qingchen Meng^2^, Wenmiao Shu^4^, Junxi Wu^4^, Liming Bian†^5,6^, Fengxuan Han†^2^, Bin Li†^1,2^**

^1^State Key Laboratory of Radiation Medicine and Protection, School of Radiation Medicine and Protection and Interdisciplinary Sciences (RAD-X), Collaborative Innovation Center of Radiation Medicine of Jiangsu Higher Education Institutions, Suzhou Medical College, Soochow University, Suzhou, Jiangsu 215006, China.

^2^Orthopedic Institute, Department of Orthopaedic Surgery, The First Affiliated Hospital, Suzhou Medical College, Soochow University, Suzhou, Jiangsu, China.

^3^Key Laboratory of Biorheological Science and Technology, Ministry of Education, College of Bioengineering, Chongqing University, Chongqing, 400044, China.

^4^Department of Biomedical Engineering, University of Strathclyde, Glasgow, G1 1QE, UK.

^5^School of Biomedical Sciences and Engineering, South China University of Technology, Guangzhou International Campus, Guangzhou 511442, People's Republic of China.

^6^National Engineering Research Center for Tissue Restoration and Reconstruction, South China University of Technology, Guangzhou 510006, P. R. China.

Correspondence should be addressed to Bin Li (binli@suda.edu.cn), Fengxuan Han ([fxhan@suda.edu.cn](mailto:fxhan@suda.edu.cn)) and Liming Bian (bianlm@scut.edu.cn).

**This PDF file includes:**

Supplementary Text

Figures S1 to S7

Tables S1

Supplementary Text

Figure S1. SEM image (A) and XRD measurement (B) of OCP.

Figure S2. QK loading efficiency of the composite hydrogels. *, *p* < 0.05.

Figure S3. SEM images of cross section of the hydrogels immersed in PBS.

Figure S4. Swelling property (A) and mass remaining (B) of the composite hydrogels immersed in PBS. *, *p* < 0.05.

Figure S5. Cell proliferation of (A) HUVECs and (B) BMSCs on the surface of composite hydrogels. *, *p* < 0.05.

Figure S6. Quantitative analysis of ALP (A) and alizarin red (B) staining. *, *p* < 0.05.

Figure S7. Bone mineral density of regenerated tissues in the bone defects.

Table S1. PCR primer sequences.


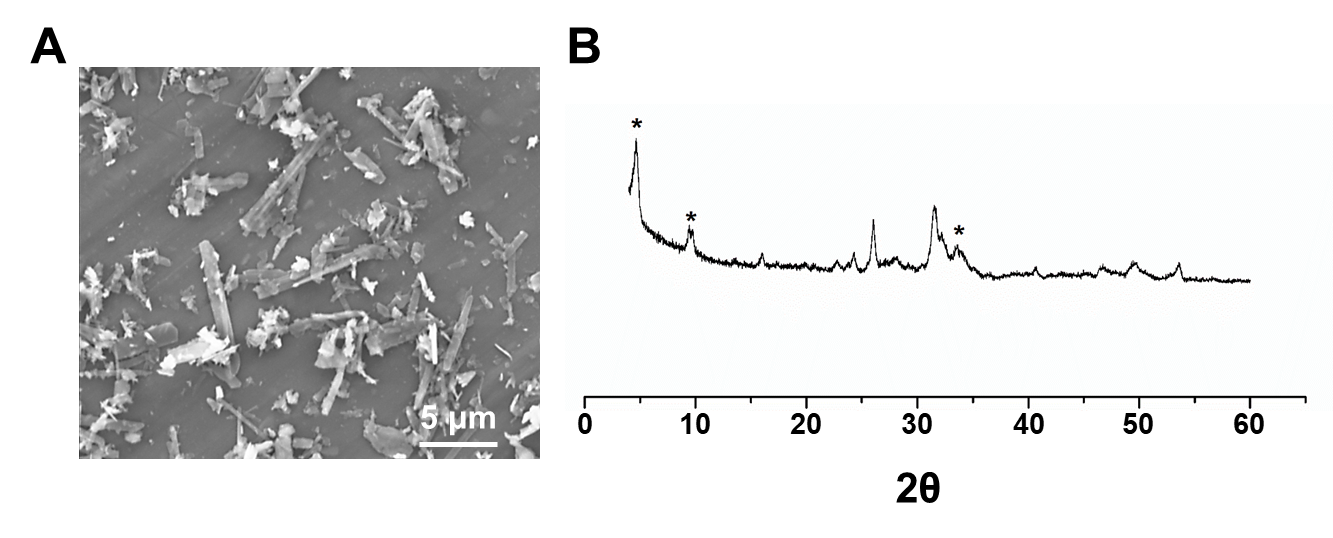


**Figure S1. SEM image (A) and XRD measurement (B) of OCP.**


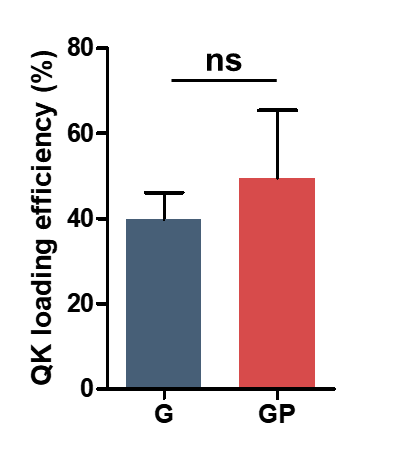


**Figure S2. QK loading efficiency of the composite hydrogels. *, *p* < 0.05.**


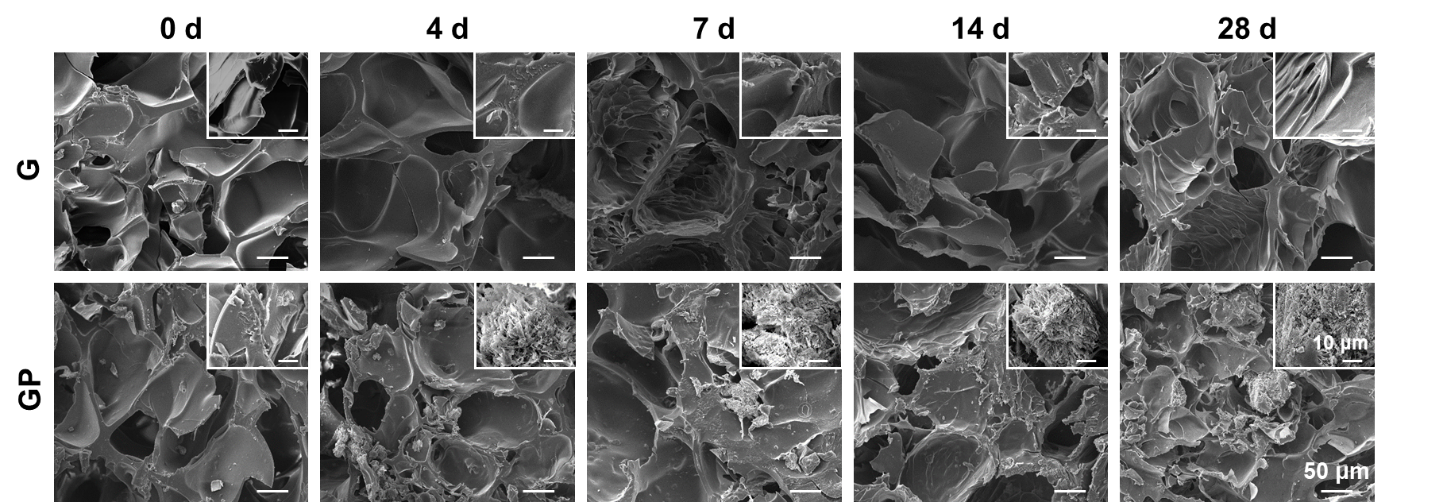


**Figure S3.** **SEM images of cross section of the hydrogels immersed in PBS.**


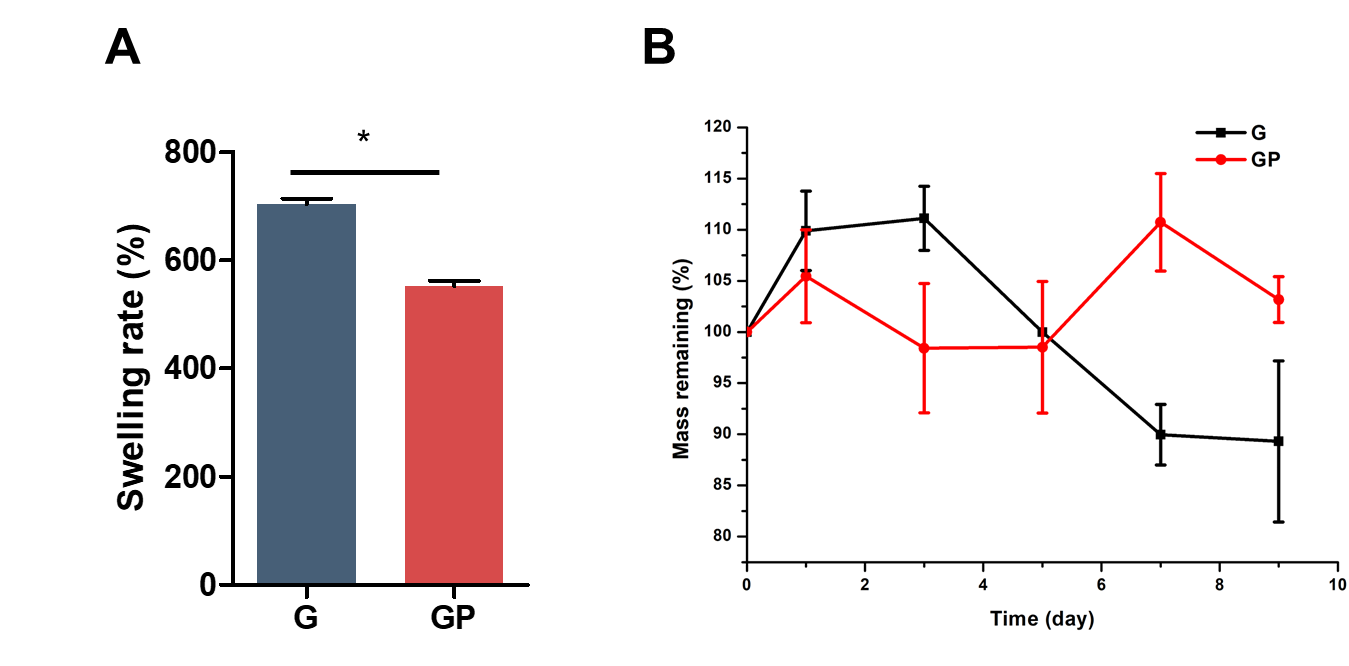


**Figure S4. Swelling property (A) and mass remaining (B) of the composite hydrogels immersed in PBS. *, *p* < 0.05.**

**
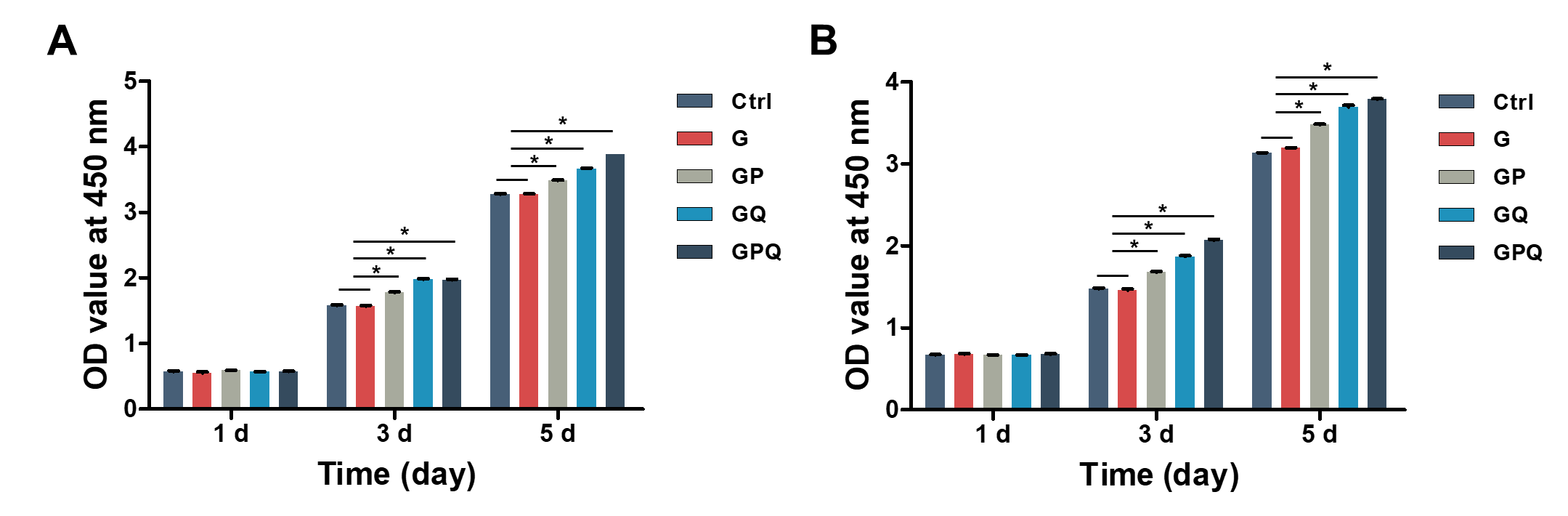
**

**Figure S5.** **Cell proliferation of (A) HUVECs and (B) BMSCs on the surface of composite hydrogels. *, *p* < 0.05.**

**
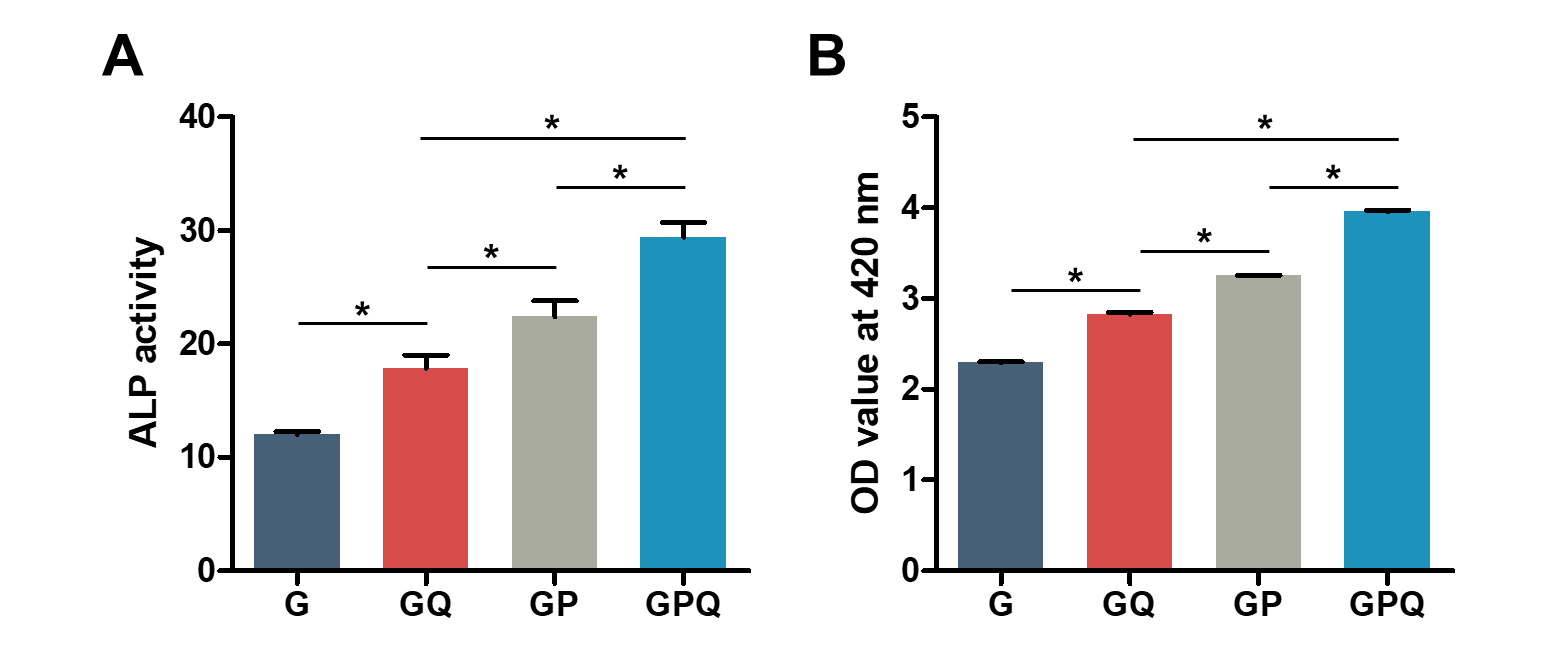
**

**Figure S6. Quantitative analysis of ALP (A) and alizarin red (B) staining. *, *p* < 0.05.**


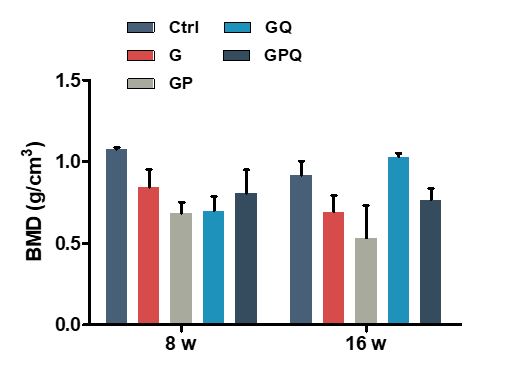


**Figure S7.** **Bone mineral density of regenerated tissues in the bone defects.**

**Table S1. PCR primer sequences.**

| Target gene | Forward primer sequence (5’–3’) | Reverse primer sequence (5’–3’) |
| --- | --- | --- |
| Alpl | TATGTCTGGAACCGCACTGAAC | CACTAGCAAGAAGAAGCCTTTGG |
| Spp1 | GCGGTTCACTTTGAGGACAC | TATGAGGCGGGGATAGTCTTT |
| Runx2 | ATCCAGCCACCTTCACTTACACC | GGGACCATTGGGAACTGATAGG |
| Col1a1 | CAGGCTGGTGTGATGGGATT | CCAAGGTCTCCAGGAACACC |
| Bglap | AACGGTGGTGCCATAGATGC | AGGACCCTCTCTCTGCTCAC |
| Flt1 | TCACCACGGACCTCAATACA | CGATGCTTCACGCTGATAAA |
| Kdr | GGAAGGTTGCTTGCTCTCAC | CAGGGCAGACAAGTGGGTAT |
| VEGF | GGCAATAGCTGCGCTGGTAGA | TGGACCCTGGCTTTACTGCTG |
| Actin | CTCATGCCATCCTGCGTCTG | GGCAGTGGCCATCTCTTGCT |
